# Identification and characterisation of a novel homozygous *KDM5A* variant associated with severe axial hypotonia, seizures, and cardiac anomalies

**DOI:** 10.1038/s10038-026-01506-6

**Published:** 2026-08-19

**Authors:** Serdar Bozlak, Cuneyd Yavas, Yusuf Seflekci, Tunay Dogan, Yunus Emre Arvas

**Affiliations:** 1 Department of Pediatric Genetics, Basaksehir Cam and Sakura City Hospital, Istanbul, Turkey; 2 Department of Molecular Biology and Genetics, Faculty of Engineering and Natural Sciences, Biruni University, Istanbul, Turkey; 3 Department of Medical Laboratory Techniques, Istinye University Vocational School of Health Care Services, Istanbul, Turkey; 4 Department of Molecular Biology and Genetics, Faculty of Science, Van Yüzüncü Yil University, Van, Turkey

**Keywords:** Neurodevelopmental disorders, Disease genetics

## Abstract

KDM5A is a critical chromatin-modifying enzyme with lysine-specific demethylase activity and is highly expressed in brain tissues. Biallelic pathogenic variants in *KDM5A* are linked to *KDM5A*-related neurodevelopmental disorders, a rare disorder involving significant cognitive and motor impairments. We conducted a clinical and genetic evaluation of a patient with severe neurodevelopmental delay. Exome sequencing was performed to identify the genetic aetiology, followed by quantitative expression analysis to assess the functional impact of the identified variant on *KDM5A* mRNA levels. A novel homozygous *KDM5A* c.2836 C > T p.(Gln946Ter) variant was identified in the proband. Quantitative analysis showed a reduction in *KDM5A* expression. However, this decrease was not statistically significant. The patients displayed neuromotor developmental delay, severe axial hypotonia, absence of head control and mild upper-extremity spasticity. Additional findings included a history of seizures, facial dysmorphism and congenital heart disease. These findings expand the known clinical spectrum of El Hayek-Chahrour neurodevelopmental disorder (OMIM #620820), specifically highlighting severe motor impairment and complex cardiac involvement, including major aortopulmonary collateral artery, as previously undescribed features of this condition. The non-significant decrease in expression suggests that pathogenicity may arise from altered protein function rather than transcript depletion. Our findings underscore the importance of exome sequencing in delineating phenotypic variability in rare neurodevelopmental syndromes.

## Introduction

The *KDM5A* gene, which encodes lysine-specific demethylase 5 A, is a 28-exon gene located at the cytogenetic locus 12p13.33 and is also referred to as retinoblastoma-binding protein 2 (*RBP2*) and Jumonji/ARID domain-containing protein 1 A (*JARID1A*) [1]. *KDM5A* is a prominent epigenetic regulator that catalyses the Fe(II)- and 2-oxoglutarate-dependent removal of di- and trimethyl marks from lysine 4 of histone H3 (H3K4me2/3), thereby reconfiguring chromatin architecture and transcriptional competence without altering the underlying DNA sequence [2]. By selectively erasing these activating histone marks, which are enriched at transcriptionally active promoters and regulatory regions, *KDM5A* fine-tunes promoter accessibility, recalibrates transcriptional thresholds and, in concert with transcription factors, chromatin-remodeling complexes and repressive complexes, facilitates the precise and hierarchical establishment of context-dependent gene expression programs [2].

The available literature indicates that *KDM5A* plays an important role in the pathogenesis of various diseases. The *NUP98::KDM5A* fusion gene, generated by a rearrangement involving nucleoporin 98 (*NUP98*) and *KDM5A*, has been implicated in the pathogenesis of acute myeloid leukaemia (AML) by promoting hematopoietic cell proliferation and perturbing myeloerythroid differentiation through aberrant epigenetic and transcriptional regulation [3–5]. Beyond AML, *KDM5A* has also emerged as a potentially important anticancer therapeutic target, as its aberrant expression in a broad range of solid tumours has been implicated in multiple hallmarks of tumour aggressiveness, including impaired differentiation, angiogenic and epithelial-mesenchymal reprogramming, enhanced invasive and metastatic capacity and therapeutic resistance; accordingly, *KDM5A* inhibitors have attracted considerable interest as targeted therapeutic agents [4, 5]. This condition is formally recognized as El Hayek-Chahrour neurodevelopmental disorder (NEDEHC; OMIM #620820), an autosomal recessive syndromic entity characterized by absent speech, intellectual disability and autism [6]. El Hayek et al., In this study, we aimed to present the clinical and molecular characteristics of our patient, in whom a novel KDM5A variant was identified and to compare these findings with those of previously reported *KDM5A*-associated cases in the literature.

## Materials and methods

### Patient and ethics

Ethical approval for this study was obtained from the Clinical Research Ethics Committee of Basaksehir Cam and Sakura City Hospital (Approval No: KAEK/126, Approval Date: 25.03.2026). The parents are first-degree cousins, indicating consanguinity within this family. Written informed consent was obtained from the participant and/or their legal guardians before inclusion. All procedures were conducted in accordance with the principles of the Declaration of Helsinki established by the World Medical Association.

In addition, bioinformatic analyses were conducted to assess the potential structural and functional impact of the identified variant. Computational tools were employed to predict changes in protein structure and function and relevant databases and the literature were reviewed for previously reported variants.

Radiological evaluation was performed using the patient’s X-ray images. The radiographic findings were systematically assessed and documented for correlation with the clinical and genetic data.

### Genetic analysis

Genomic DNA was isolated from peripheral blood samples using a commercially available extraction kit following the manufacturer’s recommendations. DNA quality and concentration were assessed spectrophotometrically and samples were preserved under appropriate storage conditions until further processing. Whole exome sequencing (WES) was performed to screen for coding region variants. Sequencing reads were aligned to the human reference genome (GRCh37/hg19) and subsequent data processing steps, including variant calling and filtering, were performed using established bioinformatics workflows. Variant coordinates are also provided accordingly. Candidate variants were prioritised based on allele frequency in population databases, previously reported evidence and predictions from multiple in silico tools. Variant classification was conducted in accordance with the ACMG 2015 Guidelines proposed by the American College of Medical Genetics and Genomics.

Secondary findings analysis was performed on the exome data to identify potentially pathogenic variants in other disease-associated genes. No additional pathogenic or likely pathogenic variants were identified in genes known to be associated with cardiac, skeletal, urogenital or endocrine phenotypes, supporting *KDM5A* as the primary molecular aetiology in this case.

To further investigate the effect of the identified variant, gene expression was analysed by quantitative real-time PCR (qPCR). Total RNA was extracted from peripheral blood samples and reverse-transcribed into complementary DNA (cDNA). Amplification reactions were prepared with a commercially available 2X qPCR master mix and run on a real-time PCR system. Each reaction was assembled in a final volume of 20 µL, including cDNA template, gene-specific forward and reverse primers and master mix (*KDM5A* gene 5’ Forward: ACTCTGGGGTAGGGTTGGC and 5’ Reverse: TGCCTCGGTCTTGCCTGTAG). The primers were designed to span the variant site (c.2836 C > T), directly encompassing the premature termination codon, to enable assessment of nonsense-mediated mRNA decay NMD status at the transcript level. Thermal cycling conditions consisted of an initial denaturation step, followed by repeated cycles of denaturation, annealing and extension under optimised conditions. approach with an internal reference gene for normalisation. The *GAPDH* gene was used as a housekeeping reference gene, with the following primers: forward, *5’-CCACCCATGGCAAATTCC-3’*; and reverse, *5’-TGGGATTTCCATTGATGACAAG-3’*. RT-qPCR was performed on RNA samples from the proband, mother and father (each *n* = 1 biological individual) and three unrelated healthy controls (*n* = 3 biological individuals), each run in three technical replicates. Given that the proband, mother and father each comprised a single biological individual, formal inferential statistical testing was not applied. Relative expression levels were determined using the comparative Ct (2^–ΔΔCt^). The control group mean was set as the reference.

Ideally, western blot analysis of patient-derived material would directly determine whether a truncated KDM5A protein is produced and at what level, as RNA-based data alone cannot distinguish between NMD-mediated transcript loss and stable production of a truncated protein. The absence of protein-level data represents a limitation of the current study. This should be addressed in future studies.

### In silico analyses

The proteins that interacted with KDM5A were identified using STRING v12.0 (https://string-db.org), with the organism set to *Homo sapiens*. The 3D structures of wild-type and mutant KDM5A were predicted using the AlphaFold server (https://alphafoldserver.com). The 3D structure of the RB1 protein was retrieved from the Protein Data Bank (https://www.rcsb.org) (PDB ID: 1GUX; Resolution: 1.85 Å).

The molecular docking analysis was performed using the HDOCK server (http://hdock.phys.hust.edu.cn). The KDM5A protein was selected as the receptor and the RB1 protein was chosen as the ligand for the docking. Then, the docked complexes were visualised and coloured using Chimaera. Additionally, interacting residues were visualised in PyMOL (https://www.pymol.org).

## Results

### Clinical findings

**Fig. 1:**
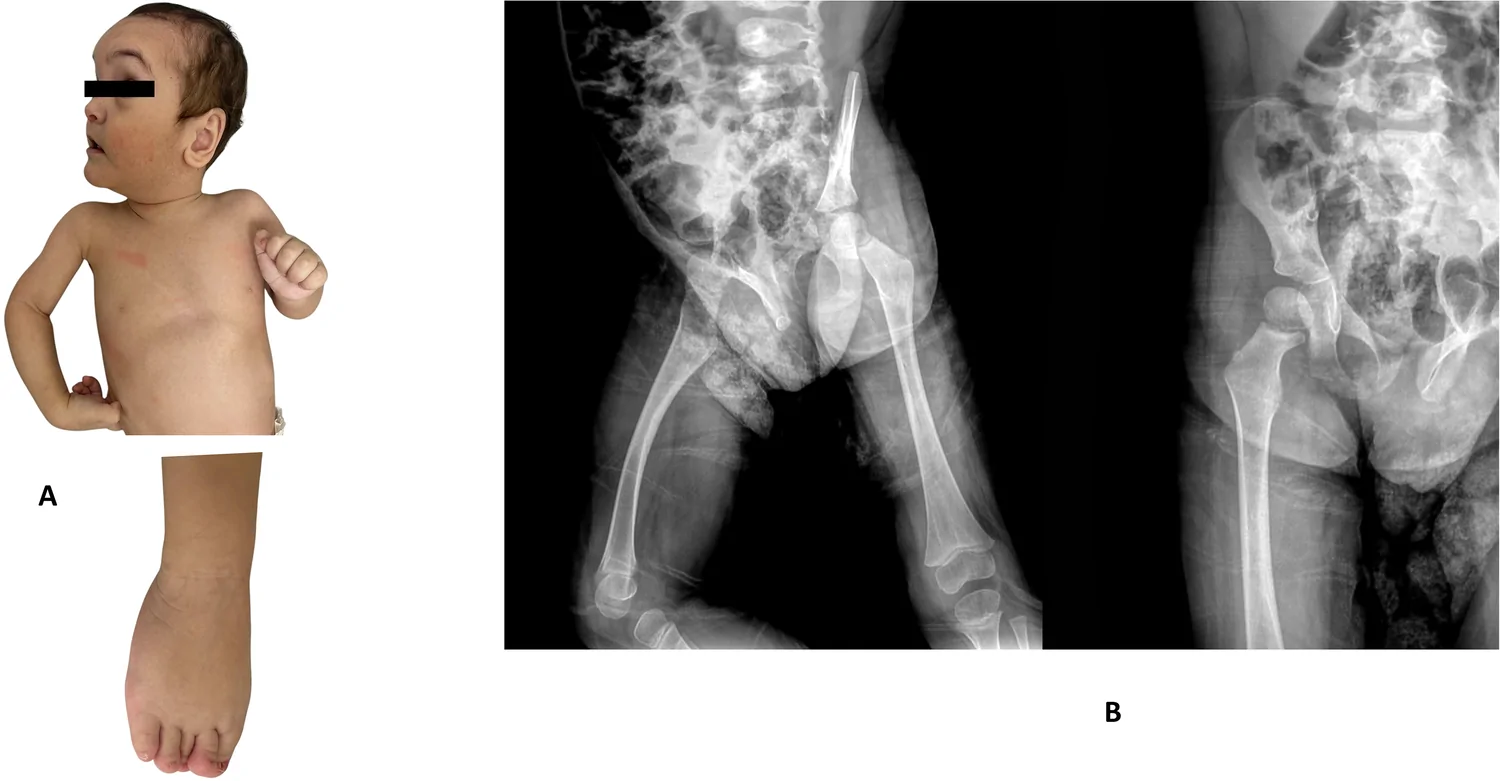
Clinical and radiological features of the patient. **A** External appearance demonstrating dysmorphic features and skeletal abnormalities, including craniofacial dysmorphism and lower extremity deformities. **B** Pelvic radiographs showing a shallow acetabulum with superolateral displacement of the femoral head, consistent with unilateral developmental dysplasia of the hip

The proband was referred to our clinic at 2 years and 2 months of age with neuromotor developmental delay and was subsequently followed up. Formal neurodevelopmental and cognitive assessment was not yet feasible at the time of evaluation, given the patient’s age of 2 years and 2 months. No words or speech attempts were documented, consistent with the absence of expressive language. Social communication and autism-specific behaviours were not formally assessed using standardised instruments (like ADOS-2, DSM-5 criteria) at the current age. These developmental domains will require longitudinal follow-up for formal characterisation. He was born at 37 weeks and 5 days of gestation with a birth weight of 2500 g (SDS: –1.3), a length of 52 cm (SDS: +1.7) and a head circumference of 32 cm (SDS: +1.47). At presentation, the head circumference was 46 cm (SDS: −2.31), representing a decline of 3.78 SD from birth, consistent with progressive postnatal microcephaly. This trajectory is clinically notable as an independent finding, distinct from the dysmorphic facial features and suggests ongoing neurological compromise during the postnatal period. Intermediate head circumference measurements between the neonatal period and the time of presentation were not available for formal growth curve analysis. The postnatal period was complicated by respiratory distress requiring admission to the neonatal intensive care unit for approximately 2.5 months. During this period, seizures developed and treatment with levetiracetam and phenobarbital was initiated. Levothyroxine therapy was also started following the diagnosis of hypothyroidism. At presentation, physical examination revealed a body weight of 10.5 kg (SDS: –1.92), a height of 89 cm (SDS: –0.28) and a head circumference of 46 cm (SDS: –2.31). There was a marked delay in neuromotor developmental milestones, accompanied by severe axial hypotonia, absent head control and mild spasticity of the upper extremities. Dysmorphic facial and skeletal features included a prominent metopic ridge secondary to premature metopic suture closure, dolichocephaly, hypertelorism, a long philtrum, long and highly arched eyebrows, long eyelashes and a high-arched palate. Pectus excavatum was also present. The ears were large (6.5 cm, >97th percentile) and the hands were small, with a hand length of 9.5 cm (<3rd percentile) (Fig. 1A). Additional examination findings included bilateral single transverse palmar creases, an overriding second toe of the right foot, rocker-bottom feet and talipes equinovarus deformities of the lower extremities. A hemangiomatous lesion on the back, congenital dermal melanocytosis, micropenis and hydrocele were also noted. The patient also had feeding difficulties and poor weight gain. Cranial magnetic resonance imaging and electroencephalography were normal and abdominal and thyroid ultrasonography findings were unremarkable. Echocardiography revealed a moderate secundum atrial septal defect, minimal mitral regurgitation and a single major aortopulmonary collateral artery (MAPCA). As the MAPCA was solitary and hemodynamically insignificant, further confirmatory imaging with CT angiography or cardiac catheterisation was not pursued. Although MAPCAs classically accompany conotruncal defects with reduced native pulmonary flow, isolated single MAPCAs without outflow tract anomalies have been rarely reported and may represent an incidental vascular developmental variant in the context of *KDM5A* haploinsufficiency, reflecting the broad cardiovascular developmental vulnerability associated with chromatin remodeling defects. The patient was placed under cardiology follow-up for these findings. Radiological evaluation, performed to investigate the clinical phenotype further, demonstrated a shallow acetabulum and superolateral displacement of the femoral head on pelvic radiographs, consistent with unilateral developmental dysplasia of the hip (Fig. 1B).

### Genetic results

**Fig. 2:**
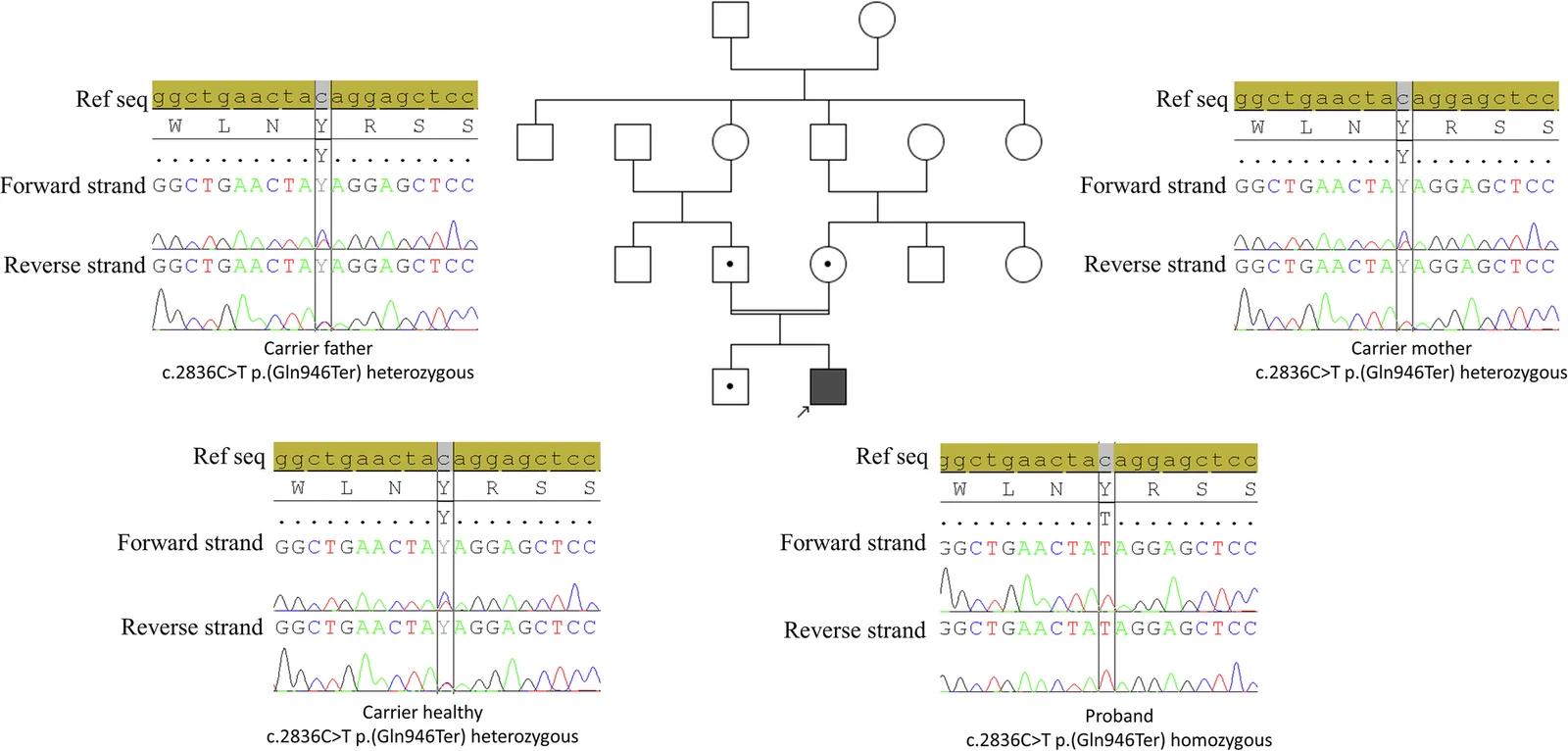
Segregation analysis of the c.2836 C > T p.(Gln946Ter) variant. Pedigree and Sanger sequencing chromatograms showing heterozygosity in both parents and the sibling and a homozygous variant in the proband, consistent with autosomal recessive inheritance

Chromosomal microarray analysis did not reveal any pathogenic copy-number variants. Whole-exome sequencing identified a homozygous nonsense novel variant, c.2836 C > T p.(Gln946Ter), in the proband. This variant is absent from the gnomAD v4 database (0 alleles observed; allele frequency = 0), supporting its classification under PM2 (absent from controls). Parental consanguinity raises the likelihood of additional homozygous regions in the proband’s genome. However, secondary findings analysis of the exome data did not reveal any additional pathogenic variants in genes associated with the proband’s phenotypic features. Segregation analysis showed that the healthy sibling was heterozygous for the variant and both parents were confirmed to be heterozygous carriers, consistent with an autosomal recessive inheritance pattern (Fig. 2).

### RNA expression profile

**Fig. 3:**
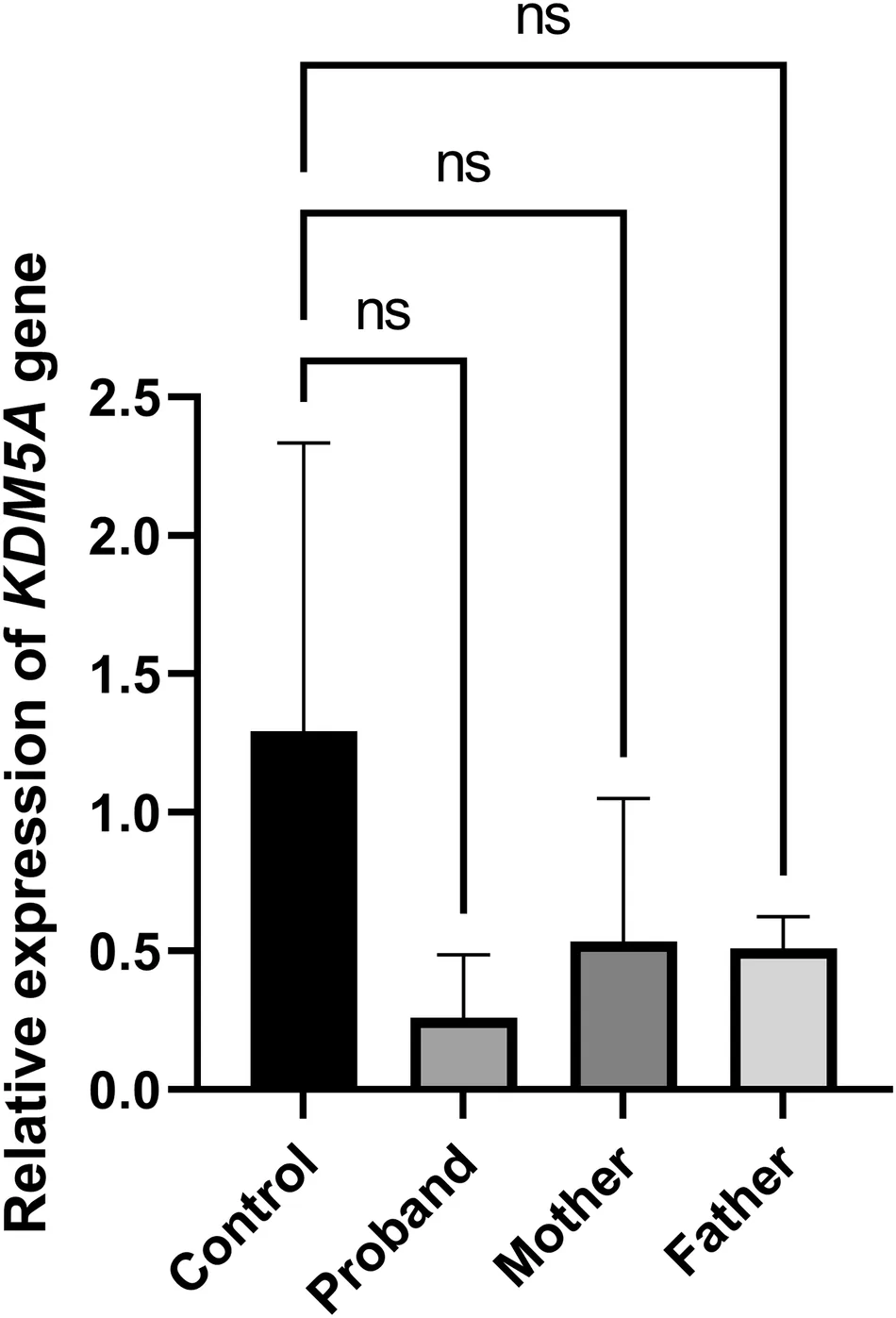
Relative expression levels of the *KDM5A* gene. RT-qPCR analysis showing relative *KDM5A* expression in the proband, parents and control. Although a slight decrease in expression was observed in the proband, no statistically significant differences were detected between the groups (ns). Data are presented as mean ± standard deviation. Data are presented as mean ± SD of three technical replicates. *n* = 1 (proband), 1 (mother), 1 (father), 3 (controls). Descriptive comparison; formal statistical testing was not applied due to single-individual sampling in patient groups. ns: no significant difference observed

RT-qPCR analysis of the *KDM5A* gene expression did not reveal any statistically significant differences among the proband, parents and control group (Fig. 3). Although a trend toward reduced expression was observed in the proband compared to the control, this difference did not reach statistical significance. Similarly, expression levels in both parents were comparable to those of the control group.

### Bioinformatic results

All structural and docking analyses presented below are computational predictions and should be regarded as hypothetical and exploratory. The results reflect potential structural consequences, assuming that a truncated protein is expressed and require experimental validation before mechanistic conclusions can be drawn.

Protein-protein interaction analysis showed that KDM5A interacts with RB1. (Supplementary file Fig. 1). The predicted structure of the wild-type KDM5A protein displays an extended multidomain architecture composed predominantly of α-helical elements connected by flexible loop regions (Supplementary file Fig. 2A). In contrast, the mutant KDM5A structure carrying the p.(Gln946Ter) variant exhibited a markedly truncated conformation. The premature stop codon results in the loss of a substantial portion of the C-terminal region of the protein, leading to the absence of several structural elements in the wild-type protein (Supplementary file Fig. 2B). Consequently, the mutant structure appears shorter and lacks the multiple secondary structural segments observed in the full-length protein (Supplementary file Fig. 2C).

**Fig. 4:**
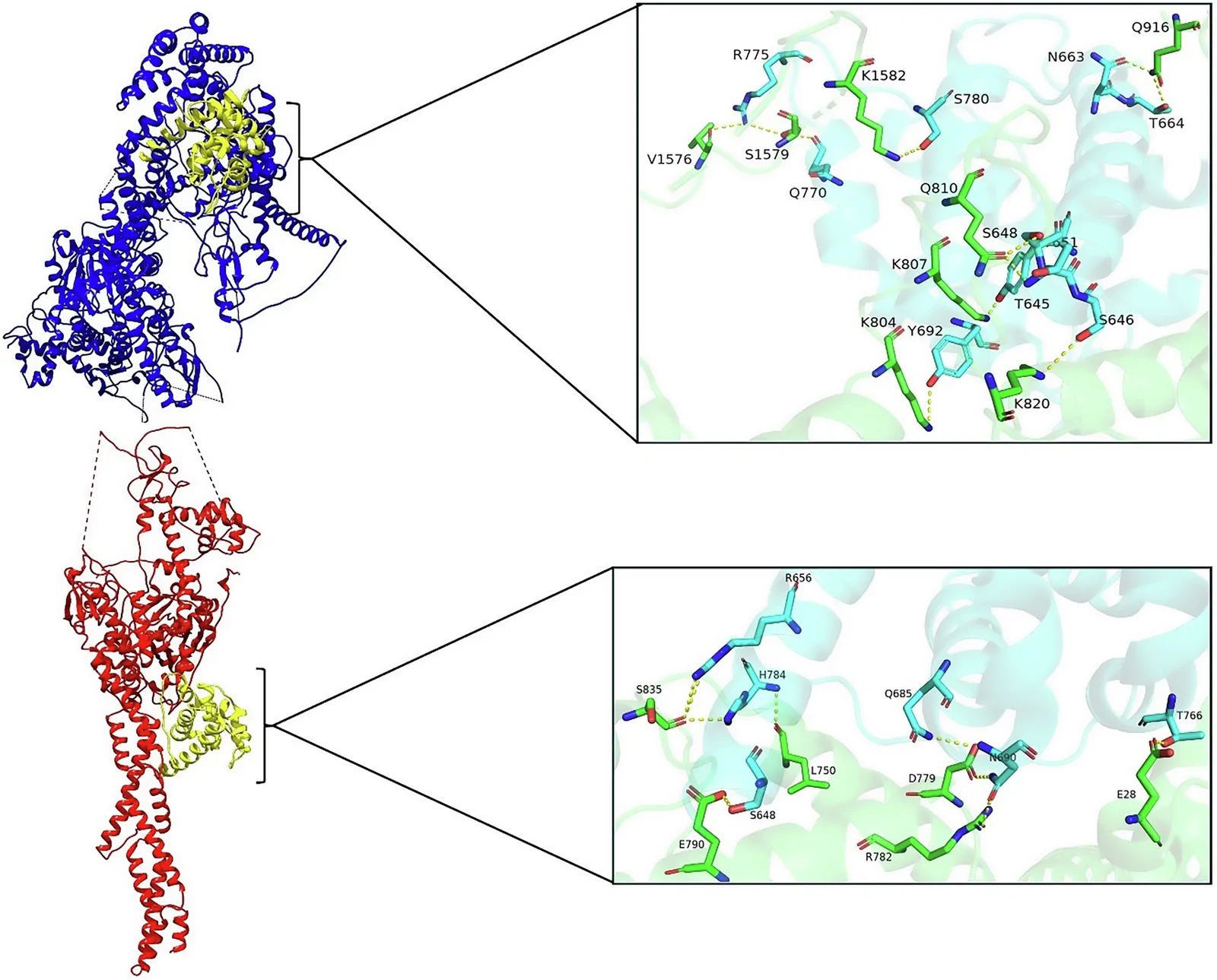
Interacted residues were identified between the wild-type and mutant forms of the KDM5A and RB1 proteins

The wild-type KDM5A protein showed a more favourable docking score with RB1 than the mutant protein, suggesting a potentially lower binding affinity of the truncated form. Furthermore, the predicted interaction interfaces differed between the wild-type and mutant complexes (Table 1; Fig. 4 and Supplementary file Fig. 1).

**Table 1:** Interacting residues identified in the docked complexes of wild-type and mutant KDM5A with RB1

Protein–protein	Docking score	Interacted residues
WT_KDM5A_RB1	-280.08	V1576-R775; S1579-R775; S1579-Q770; K1582-S780; Q916-N663; Q916-T664; Q810-S648; Q810-T645; K807-Y651; K820-S646; K804-Y692
MT_KDM5A_RB1	-248.69	E28-T766; R782-N690; D779-N690; D779-Q685; E790-S648; L750-H784; S835-S656; S835-H784

*WT_KDM5A: Wild type KDM5A; **MT_KDM5A_ RB1**: Mutant type KDM5A; **RB1**: Retinoblastoma 1

## Discussion

In silico analysis suggests that the p.(Gln946Ter) variant of *KDM5A* may reduce binding affinity with RB1 and is predicted to alter the binding interface (Table 1; Fig. 3). KDM5A contains the LxCxE motif between the PHD2 and PHD3 fingers, which is responsible for its interaction with various pocket proteins such as RB, p107 and p130 [2]. While wild-type KDM5A contains PHD2 and PHD3, the mutant form lacks these domains due to the variant. Therefore, this provides a potential structural basis for the predicted lower docking score and altered interaction interface of the mutant relative to the wild type.

However, this variant is predicted to result in a truncated protein and its expression and stability cannot be assumed without experimental validation. Although NMD eliminates transcripts carrying premature termination codons, previous studies have shown that some transcripts can escape this mechanism and give rise to truncated proteins with variable stability [7, 8]. Therefore, the presented in silico results should be interpreted as hypothetical and exploratory, reflecting potential structural consequences under the assumption that a truncated protein is expressed.

Until relatively recently, little was known about the relationship between *KDM5A* and neurodevelopmental disorders. To the best of current knowledge, the first report was published by Najmabadi et al. in 2011, who described a recessive missense variant in *KDM5A* in a patient with an undefined developmental disorder (Table 2) [9]. Subsequently, Silva et al. reported a patient with autism spectrum disorder (ASD) harbouring an approximately 1.5-Mb terminal deletion at 12p13.33 encompassing *KDM5A*; however, given the multigenic nature of the deletion, they suggested that *ERC1*, rather than *KDM5A*, might represent the principal candidate gene contributing to the neurodevelopmental delay and autistic phenotype [10]. In subsequent years, additional patients harbouring terminal 12p13.33-p13.32 microdeletions encompassing *KDM5A* were also reported, including a Korean family with epilepsy, intellectual disability and schizophrenia, in whom *KDM5A* was considered one of the candidate genes potentially contributing to the neuropsychiatric phenotype within the multigenic deleted interval [11].

**Table 2:** Reported *KDM5A* variants and clinical findings in previously published cases and in our case

PN	G/A	Neurological and Neuromuscular Findings	Cardiac Findings	Additional Clinical Findings	Transcript and Variant Nomenclature (c.DNA/Protein)	H	Variant Type	Z	ACMG Classification	Reference
P1	NR/NR	Intellectual disability	NR	Facial dysmorphism	NR/p.(Arg719Gly)	AR	Missense	HM	VUS(PM2,PP3)	Najmabadi et al. [9]
P2	M/NR	NR	Tetralogy of Fallot, Patent foramen ovale, Ventricular septal defect	NR	(NM_001042603.2) c.4399 C > T/p.(Arg1467Trp)	AD	Missense	HT	VUS(PM2,PP3)	Szot et al. [12]
P3	M/5	Autism spectrum disorder, Absent speech, Intellectual disability, Developmental delay, Seizures, Motor impairment, Muscle hypotonia	Heart murmur	Feeding difficulties	(NM_001042603) c.1 A > T/p.(Met1Leu)	de novo	Missense	HT	LP (PVS1,PS4,PM2)	El Hayek et al. [6]
P4	F/12	Autism spectrum disorder, Absent speech, Intellectual disability, Developmental delay, Seizures, Motor impairment, Muscle hypotonia, Hypoplastic corpus callosum	Atrial septal defect	Feeding difficulties, Facial dysmorphism	(NM_001042603) Deletion of exons 6 through 9	AR	CNV (Multi-exon deletion)	HM	Uncertain(0.45) [1 A,2A-2E,3 A]	El Hayek et al. [6]
P5	M/8	Autism spectrum disorder, Absent speech, Intellectual disability, Developmental delay, Motor impairment, Muscle hypotonia, Hypoplastic corpus callosum, Mild hippocampal atrophy	NR	Feeding difficulties, Facial dysmorphism	(NM_001042603) Deletion of exons 6 through 9	AR	CNV (Multi-exon deletion)	HM	Uncertain(0.45) [1 A,2A-2E,3 A]	El Hayek et al. [6]
P6	M/18	Autism spectrum disorder, Absent/limited speech, Intellectual disability, Developmental delay, Seizures	Ventricular septal defect	Feeding difficulties, Facial dysmorphism	(NM_001042603) c.4283 G > T/p.(Arg1428Leu)	de novo	Missense	HT	LP(PS4,PM2)	El Hayek et al. [6]
P7	F/3	Autism spectrum disorder, Absent speech, Intellectual disability, Motor impairment	NR	NR	(NM_001042603) c.2541+1 G > T	AR	Splice-site	HM	P (PVS1,PM2,PP5)	El Hayek et al. [6]
P8	M/20	Autism spectrum disorder, Absent speech, Intellectual disability, Developmental delay, Motor impairment, Muscle hypotonia	NR	Feeding difficulties	(NM_001042603) c.2541+1 G > T	AR	Splice-site	HM	P (PVS1,PM2,PP5)	El Hayek et al. [6]
P9	F/4	Autism spectrum disorder, Absent speech, Intellectual disability, Developmental delay, Motor impairment, Muscle hypotonia, Periventricular leukomalacia	Atrial septal defect	Feeding difficulties, Facial dysmorphism	(NM_001042603) c.1429 T > G/p.(Phe477Val)	AR	Missense	HM	VUS (PM2,PP3,PP5)	El Hayek et al. [6]
P10	F/40	Intellectual disability, Absent speech (few words), Developmental delay, Seizures, Motor impairment, Microcephaly, Gliosis of the parieto-central pyramidal tracts with mild parietal atrophy	NR	Facial dysmorphism	NM_001042603 c.4048 C > T/p.(Arg1350Ter)	de novo	Nonsense	HT	P (PVS1,PM2,PP5)	El Hayek et al. [6]
P11	F/13	Intellectual disability, Developmental delay, Absent speech, Motor impairment, Muscle hypotonia	Heart murmur, Coarctation of the aorta	Facial dysmorphism	NM_001042603 Deletion of exons 1 through 10	AR	CNV (Multi-exon deletion)	HM	Uncertain (0.45) [1 A,2A-2E,3 A]	El Hayek et al. [6]
P12	M/4	Autism spectrum disorder, Limited speech (few words), Intellectual disability, Developmental delay, Seizures	None	Selective eating, Sleep problems, Hand flapping	(NM_001042603) c.2414 A > G/p.(Lys805Arg)	de novo	Missense	HT	P II (PM2, BP1, PS3, PM1, PS2)	El Hayek et al. [16]
P13	M/29	Autism spectrum disorder, Absent speech, Intellectual disability, Developmental delay, Muscle hypotonia	None	Panic attacks, Bilateral 2-3 toe syndactyly	(NM_001042603) c.3347 G > A (P); c.4466 T > C (M)/p.(Gly1116Glu) (P); p.(Met1489Thr) (M)	AR	Missense	Comp. Het	LB; VUS	El Hayek et al. [16]
P14	F/spontaneous intrauterine demise at 33 1/7 weeks	Dandy-Walker malformation spectrum with hydrocephalus	Complex congenital heart disease	Facial dysmorphism, Progressive fetal macrocephaly, Polyhydramnios late in pregnancy	(NM_001042603) c.2325 C > A/p.(Tyr775Ter)	de novo	Nonsense	HT	P 1a (PM2, PVS1, PS2)	El Hayek et al. [16]
P15	F/5	Autism spectrum disorder, Absent speech, Developmental delay, Seizures, Muscle hypotonia	Murmur (resolved)	Feeding difficulties, Sleep problems, Facial dysmorphism, Hyperextensible joints, Mild nearsightedness	(NM_001042603) c.4557 A > T/p.(Glu1519Asp)	de novo	Missense	HT	LP II (PM2, BP1, PS2)	El Hayek et al. [16]
P16	F/10	Autism spectrum disorder, Delayed speech, Intellectual disability, Developmental delay, Seizures	Mild atrial septal defect	Feeding difficulties, Sleep problems, Facial dysmorphism, Self-injurious behavior, ADHD, Mania, Mood disorder, OCD, Mild common iliac artery	(NM_001042603) c.3197 C > G (P); c.3974 T > C (M)/p.(Ser1066Cys) (P); p.(Val1325Ala) (M)	AR	Missense	Comp. Het	VUS; VUS	El Hayek et al. [16]
P17	M/5	Autistic features, Absent speech, Intellectual disability, Developmental delay, Seizures, Muscle hypotonia, Thin corpus callosum, Slightly prominent lateral ventricles, Reduced white matter volume	Murmur (resolved)	Hyperextensible joints, Feeding difficulties, Facial dysmorphism, Cortical visual impairment, Solitary mastocytoma on left knee, Recurrent upper respiratory tract infections	(NM_001042603) c.1045 C > T (P); c.2427-17 A > G (M)/p.(Arg349Ter) (P); p.(Arg809Serfs*10) (M)	AR	Nonsense; Splicing	Comp. Het	P 1b; P 1a	El Hayek et al. [16]
P18	F/3 y 6 months	Absent speech, Intellectual disability, Developmental delay, Seizures, Muscle hypotonia, Mild cerebral atrophy	None	Hyperextensible joints, Feeding difficulties, Facial dysmorphism, Attention deficits, Inability to walk	(NM_001042603) c.1495 G > T/p.(Glu499Ter)	AR	Nonsense	HM	P 1c (PM2, PVS1, PP3)	El Hayek et al. [16]
P19	M/6 y 10 months	Autism spectrum disorder, Absent speech, Intellectual disability, Developmental delay, Seizures, Muscle hypotonia, Mild frontoparietal cortical atrophy, Deep Sylvian fissures, Thin corpus callosum, Mildly dilated lateral ventricles	None	Hyperextensible joints, Feeding difficulties, Sleep problems, Facial dysmorphism, Attention deficits, Constipation	(NM_001042603) c.4960 A > G/p.(Ile1654Val)	AR	Missense	HM	VUS (PM2, BP1, PM1)	El Hayek et al. [16]
P20	F/17	Absent speech, Intellectual disability, Developmental delay, Muscle hypotonia	None	Inability to walk	(NM_001042603) c.82 G > T/p.(Glu28Ter)	AR	Nonsense	HM	P 1c (PM2, PVS1, PP3)	El Hayek et al. [16]
P21	M/4	Absent speech, Intellectual disability, Developmental delay, Muscle hypotonia	None	Hyperextensible joints, Feeding difficulties, Sleep problems, Facial dysmorphism, Attention deficits, Conductive hearing loss, Constipation	(NM_001042603) c.2465 C > G/p.(Thr822Arg)	Unconfirmed	Missense	HT	VUS (PM2, BP1, PM1)	El Hayek et al. [16]
P22	F/24	Speech issues	None	ADHD, Depression, Bipolar disorder	(NM_001042603) c.2902del/p.(Arg968Glyfs*7)	AD	Frameshift	HT	P 1a (PM2, PVS1, PP3, PS3, PP1)	El Hayek et al. [16]
P23	M/3	Autism spectrum disorder, Delayed speech (few words), Developmental delay	None	Feeding difficulties, Fine motor delays, Congenital laryngomalacia, Astigmatism	(NM_001042603) c.2902del/p.(Arg968Glyfs*7)	AD	Frameshift	HT	P 1a (PM2, PVS1, PP3, PS3, PP1)	El Hayek et al. [16]
P24	M/11	Autistic features, Absent speech, Developmental delay	None	Hyperactivity	(NM_001042603) c.2002C>T/p.(Leu668Phe)	de novo	Missense	HT	LP II (PM2, PP3, BP1, PS3, PM1)	El Hayek et al. [16]
P25	M/7	Autism spectrum disorder, Absent speech, Intellectual disability, Developmental delay	None	Sleep problems, ADHD, Anxiety, Bipolar disorder, Marcus Gunn phenomenon	(NM_001042603) c.4025 A > G/p.(Glu1342Gly)	de novo	Missense	HT	P II (PM2, PP3, BP1, PS3, PS2)	El Hayek et al. [16]
P26	F/8	Autism spectrum disorder, Absent speech, Intellectual disability, Developmental delay	Peripheral pulmonary stenosis (resolved)	Feeding difficulties, Sleep problems, Constipation	(NM_001042603) c.4576 G > T/p.(Glu1526Ter)	de novo	Nonsense	HT	P 1a (PM2, PVS1, PP3, PS2)	El Hayek et al. [16]
P27	M/5 y 7 months	Autistic features, Delayed speech, Intellectual disability, Developmental delay, Muscle hypotonia, Persistent cavum septum pellucidum et vergae	None	Hyperextensible joints, Feeding difficulties, Facial dysmorphism, Attention deficits, Recurrent infections	(NM_001042603) c.901 C > T/p.(Arg301Trp)	de novo	Missense	HT	VUS (PM2, PP3, BP1, PM1)	El Hayek et al. [16]
P28	M/4 y 5 months	Autism spectrum disorder, Absent speech, Intellectual disability, Developmental delay, Muscle hypotonia, Hypoplastic temporal lobe, Deep Sylvian fissures, Thin dysplastic corpus callosum, Increased extra-axial cerebrospinal fluid in the frontoparietal regions, Deep white matter signal abnormalities, Periventricular leukomalacia around the occipital horns	Atrial and ventricular septal defects, Murmur	Hyperextensible joints, Facial dysmorphism, Attention deficits, Inability to walk, Recurrent upper respiratory tract infections, Constipation	(NM_001042603) c.796 C > T/p.(Arg266Ter)	AR	Nonsense	HM	P 1c (PM2, PVS1, PP3)	El Hayek et al. [16]
P29	F/13 y 3 months	Autistic features, Speech issues, Intellectual disability, Developmental delay, Muscle hypotonia, Reduced brain volume, Periventricular white matter changes, Mild enlargement of the cerebrospinal fluid spaces, Small pituitary gland	Aortic arch variant	Hyperextensible joints, Feeding difficulties, Facial dysmorphism, Multiple vision issues, Hearing loss	(NM_001042603) c.3860 C > T/p.(Ala1287Val)	AR	Missense	HM	VUS (PP3, BP1, PP1)	El Hayek et al. [16]
P30	F/9 y 9 months	Autism spectrum disorder, Absent speech, Intellectual disability, Developmental delay, Muscle hypotonia	Atrial and ventricular septal defects, Pulmonary hypertension	Hyperextensible joints, Feeding difficulties, Sleep problems, Facial dysmorphism, Self-injurious behavior, Hyperopia	(NM_001042603) c.3860 C > T/p.(Ala1287Val)	AR	Missense	HM	VUS (PP3, BP1, PP1)	El Hayek et al. [16]
P31	M/2 y 4 months	Autism spectrum disorder, Absent speech, Intellectual disability, Developmental delay	None	Feeding difficulties, Sleep problems, Facial dysmorphism, Self-injurious behavior, Constipation	(NM_001042603) c.3158 G > A/p.(Trp1053Ter)	de novo	Nonsense	HT	P 1a (PM2, PVS1, PP3, PS3, PS2)	El Hayek et al. [16]
P32	M/3 y 11 months	Delayed speech, Developmental delay	Right ventricular conduction delay	Hyperactivity, Aggressive behaviors	(NM_001042603) c.4564del/p.(Gln1522Argfs*6)	de novo	Frameshift	HT	P 1a (PM2, PVS1, PP3, PS2)	El Hayek et al. [16]
P33	M/1 y 3 months	Absent speech, Intellectual disability, Developmental delay, Seizures, Muscle hypotonia, Mild dilation of the subarachnoid spaces	None	Feeding difficulties, Sleep problems, Gastroenteritis	(NM_001042603) c.1308+7 A > G/p.0	AR	Splicing	HM	P 1c (PM2, PVS1, PP3)	El Hayek et al. [16]
P34	M/13	Autism spectrum disorder, Absent speech, Intellectual disability, Developmental delay, Seizures, Muscle hypotonia, Thin corpus callosum, Mildly dilated lateral ventricles	None	Hyperextensible joints, Feeding difficulties, Sleep problems, Facial dysmorphism, Hyperactivity	(NM_001042603) c.4049 G > A/p.(Arg1350Gln)	AR	Missense	HM	VUS (PM2, PP1)	El Hayek et al. [16]
P35	F/10	Autism spectrum disorder, Absent speech, Intellectual disability, Developmental delay, Seizures, Muscle hypotonia, Thin corpus callosum	None	Hyperextensible joints, Feeding difficulties, Sleep problems, Facial dysmorphism	(NM_001042603) c.4049 G > A/p.(Arg1350Gln)	AR	Missense	HM	VUS (PM2, BP1, PP1)	El Hayek et al. [16]
P36	M/2	Neuromotor developmental delay, History of seizures, Severe axial hypotonia, Absence of head control, Mild upper-extremity spasticity	Moderate secundum atrial septal defect, Minimal mitral regurgitation, Major aortopulmonary collateral artery	Facial dysmorphism, Feeding difficulties, Hypothyroidism, Pectus excavatum, Talipes equinovarus, Rocker-bottom feet, Overriding second toe, Developmental dysplasia of the hip, Micropenis, Hydrocele, Hemangioma	(NM_001042603.3) c.2836 C > T/p.(Gln946Ter)	AR	Nonsense	HM	LP (PVS1, PM2)	Our case

*PN* patient number, *G/A* gender/age, *M* male, *F* female, *H* hereditary pattern, *Z* zygosity, *HM* homozygous, *HT* heterozygous Comp. *Het* compound heterozygous, *AR* autosomal recessive, *AD* autosomal dominant, *CNV* copy number variation, *VUS* variant of uncertain significance, *LB* likely benign, *LP* likely pathogenic, *P* pathogenic, *NR* not reported, *None* absent finding, *ASD* autism spectrum disorder, *ADHD* attention-deficit/hyperactivity disorder, *OCD* obsessive-compulsive disorder, *ACMG* American College of Medical Genetics and Genomics

In 2018, Szot et al. reported a segregating heterozygous *KDM5A* missense variant (Table 2) in a family with congenital heart disease characterised by a patent foramen ovale, tetralogy of Fallot and a ventricular septal defect. They suggested that, together with a co-occurring KMT2C variant, it might contribute to cardiac maldevelopment through disturbed histone methylation. However, the finding was ultimately classified as a variant of uncertain significance (VUS) [12]. Compared with *KDM5A*, substantially more evidence is available regarding the involvement of other *KDM5* family members in neurodevelopmental disorders, particularly *KDM5B* and *KDM5C*. By 2020, El Hayek et al. had provided the clearest evidence linking *KDM5A* to ASD, a clinically heterogeneous group of neurodevelopmental conditions; using N-ethyl-N-nitrosourea-based forward genetic screening and a targeted *KDM5A* knockout mouse model (*KDM5A*^-/-^), they showed that *KDM5A* deficiency disrupts multiple neurodevelopmentally relevant domains, including vocalization, social behaviour, cognitive performance, dendritic morphogenesis and hippocampal transcriptomic homeostasis and subsequently extended these experimental observations to the clinical setting by identifying pathogenic *KDM5A* variants in nine affected individuals from seven families (Table 2), most of whom presented with ASD, absent or markedly limited speech, intellectual disability and developmental delay [13].

Gene expression studies following *KDM5A* gene variants are currently limited in the literature. Gene expression levels have been reviewed in a recent study [14]. However, they have not been investigated in patients presenting with neurodevelopmental disorders accompanied by hypotonia, dysmorphic features and multisystem involvement. In the present study, the KDM5A gene expression profile in individuals carrying the c.2836 C > T p.(Gln946Ter) variant did not differ significantly from that of the control group. Although a slight decrease in gene expression was observed in the proband, this difference was not significant and similar expression levels were detected in the parents. Clinically, the proband exhibited a complex phenotype characterised by neurological involvement, including neuromotor developmental delay, seizures, severe axial hypotonia, absence of head control and mild upper-extremity spasticity. Cardiovascular findings included a moderate secundum atrial septal defect, minimal mitral regurgitation and a major aortopulmonary collateral artery. Dysmorphic and skeletal abnormalities comprised facial dysmorphism, pectus excavatum, talipes equinovarus, rocker-bottom feet, overriding second toe and developmental dysplasia of the hip. Additional features included feeding difficulties, hypothyroidism, micropenis, hydrocele and cutaneous findings such as hemangioma. These findings suggest that the c.2836 C > T p.(Gln946Ter) variant in the *KDM5A* gene may not exert its pathogenic effect through significant alterations in gene expression and that alternative molecular mechanisms may underlie the observed clinical phenotype.

Building on their 2020 findings, El Hayek et al. further showed in a 2023 animal study that loss of *Kdm5a* in mice disrupts the establishment of hippocampal cell identity by altering early differentiation programs in distinct excitatory and inhibitory neuronal subtypes, thereby providing mechanistic support for the role of *KDM5A* in neurodevelopment [15]. These convergent experimental and clinical observations subsequently led to the curation of *KDM5A* in Online Mendelian Inheritance in Man (OMIM) as the gene associated with El Hayek-Chahrour neurodevelopmental disorder, also referred to as neurodevelopmental disorder with absent speech, impaired intellectual development and autism (NEDEHC) (OMIM #620820), a distinct syndromic neurodevelopmental entity characterized predominantly by absent speech, impaired intellectual development and autism. According to the OMIM Clinical Synopsis (OMIM #620820), El Hayek-Chahrour neurodevelopmental disorder is an autosomal recessive (AR) syndromic neurodevelopmental condition characterized primarily by impaired intellectual development, developmental delay, absent speech, hypotonia, motor impairment and autism spectrum disorder (ASD), with variable accompanying craniofacial, cardiac, skeletal and neuroimaging abnormalities, including microcephaly, low-set ears, atrial septal defect, fifth-finger clinodactyly, hypoplastic corpus callosum and periventricular leukomalacia.

It should be noted that not all previously reported patients included in Table 2 carry variants classified as pathogenic or likely pathogenic; several (including P1, P2 and others) carry variants of uncertain significance (VUS) and P2 harbours a monoallelic heterozygous variant with a potentially different mechanistic basis from the biallelic loss-of-function (LoF) cases. Phenotypic comparisons across this heterogeneous group should therefore be interpreted cautiously and severity claims are most robustly supported when restricted to the biallelic LoF subgroup. Reported *KDM5A* cases reveal that the inheritance pattern of *KDM5A*-related neurodevelopmental disorders has not yet been fully established; both monoallelic (heterozygous, including de novo) and biallelic (homozygous) variants have been documented in affected individuals, underscoring the genetic complexity of this emerging condition. Notably, biallelic loss-of-function variants (such as the homozygous splice-site variant (c.2541+1 G > T) shared by P7 and P8, the multi-exon deletions in P4, P5 and P11 and the de novo nonsense variant (c.4048 C > T/p.(Arg1350Ter) in P10) consistently associate with a more severe and multisystem phenotype. In contrast, monoallelic de novo variants tend to present with a predominantly neurobehavioral profile [9, 12, 13]. Against this backdrop, our patient (P12) harbours a novel homozygous nonsense variant (NM_001042603.3: c.2836 C > T/p.(Gln946Ter)), which introduces a premature stop codon predicted to result in nonsense-mediated mRNA decay or a severely truncated non-functional protein and is classified as Likely Pathogenic (PVS1, PM2) per ACMG/AMP criteria. Segregation analysis confirmed heterozygosity in both parents and the unaffected sibling. However, because only one affected individual was available in this family, PP1 (co-segregation with disease) was not applied at full strength and was therefore not included in the final classification. To the best of our knowledge, this is the thirty-fifth reported patient with a pathogenic or likely pathogenic *KDM5A* variant. This is the first Turkish individual reported to have a likely pathogenic KDM5A variant and represents an independent report from outside the Chahrour group. Since the 2020 series [8], El Hayek et al. have further expanded the *KDM5A* variant spectrum in a 2026 cohort comprising 24 additional individuals from 21 families [16], bringing the total number of reported cases to over 35. Our patient adds to this growing body of evidence and contributes a distinct phenotypic profile not previously described within this cohort. Phenotypically, our patient shares core neurodevelopmental features with previously reported biallelic cases -including developmental delay, seizures, hypotonia and cardiac anomalies- yet exhibits a distinctly more severe motor phenotype characterized by complete absence of head control and the most complex cardiac involvement reported to date among biallelic cases, comprising moderate secundum atrial septal defect, minimal mitral regurgitation and a major aortopulmonary collateral artery, a finding not previously described in any *KDM5A* patient [12, 13].

A particularly informative comparison can be drawn between our patient and those reported to carry LoF variants (specifically nonsense, frameshift and truncating variants) across all three KDM5 paralogs, as such variants are predicted to result in the most severe degree of protein depletion through NMD and therefore represent the closest mechanistic analogue to the homozygous nonsense variant identified in our patient (p.(Gln946Ter)). Within the *KDM5B* literature, the cohort described by Faundes et al. [17]. constitutes the most directly comparable group: three unrelated males carrying homozygous or compound heterozygous *KDM5B* frameshift and nonsense variants all demonstrated moderate-to-severe intellectual disability and developmental delay, with feeding difficulties, mild growth failure and dysmorphic facial features, including camptodactyly of the fourth and fifth fingers [17]. Notably, none of these patients were reported to have cardiac anomalies, seizures or hypotonia, distinguishing them phenotypically from our patient despite the shared LoF mechanism [18]. The broader cohort of Borroto et al. [19] encompassing 21 individuals with various *KDM5B* variant types, confirmed that the most common features across both dominant and biallelic groups are neurodevelopmental delay, intellectual disability, autistic behaviors, sleep disorders and facial dysmorphism; however, cardiac anomalies remained conspicuously absent and the degree of motor impairment was markedly less severe than that observed in our patient.

Within the *KDM5C* series, truncating and LoF variants have been more extensively characterised owing to the larger number of reported patients. Wu et al. [20] reported two unrelated families carrying novel hemizygous *KDM5C* variants (a missense p.(Gln745Glu) and a frameshift p (Glu1131 AlafsTer72)) in which affected males presented with severe intellectual disability, epilepsy, autism spectrum disorder and spastic paraplegia, with one patient additionally demonstrating partial agenesis of the corpus callosum. The frameshift variant in this series, predicted to result in NMD, was associated with the more severe phenotypic profile, including epilepsy and ASD—features consistent with the observation that truncating *KDM5C* variants tend to correlate with greater clinical severity than missense variants, particularly when located outside functional domains [20]. Lintas et al. [21]. Reported a de novo heterozygous nonsense variant in *KDM5C* (p.(Glu1283Ter)) in a female patient with moderate intellectual disability and neuropsychiatric features, demonstrating that even heterozygous LoF variants in *KDM5C* can produce a clinically significant phenotype in females. The large meta-analysis by Terry et al. [22] further confirmed that seizures occur in over one-third of individuals with *KDM5C* variants regardless of sex and that growth abnormalities, vision impairment and digestive issues are also recurrent features, emphasising the multisystem nature of *KDM5C*-related neurodevelopmental disorder.

Taken together, these cross-paralog comparisons highlight several phenotypic themes that are specifically amplified in our patient (P12) relative to other *KDM5* LoF cases. While intellectual disability, developmental delay and feeding difficulties are shared across *KDM5A*, *KDM5B* and *KDM5C* LoF carriers, the severity of motor dysfunction in P12 (manifesting as complete absence of head control and severe axial hypotonia) exceeds what has been reported in biallelic *KDM5B* patients [17, 18] and approaches the upper end of the severity spectrum described in hemizygous *KDM5C* males [20], suggesting that complete biallelic loss of *KDM5A* may exert a disproportionate impact on the developing motor system. Cardiac anomalies have not been reported in published *KDM5B* or *KDM5C* series; however, systematic echocardiographic screening was not uniformly performed in these comparator cohorts and the apparent absence of cardiac findings may reflect ascertainment bias rather than a true biological difference. With this important caveat in mind, the possibility that *KDM5A* may have a paralog-specific role in cardiac morphogenesis that KDM5B or KDM5C does not compensate for remains an intriguing hypothesis that warrants investigation in larger cohorts and animal models. The structural cardiac anomaly in P12, namely the MAPCA, has not been previously described in any *KDM5* family member and its co-occurrence with an atrial septal defect and mitral regurgitation in a patient with biallelic *KDM5A* LoF further supports this hypothesis. The multisystem involvement observed in P12 (including hypothyroidism, skeletal deformities (pectus excavatum, talipes equinovarus, rocker-bottom feet, developmental dysplasia of the hip), urogenital anomalies (micropenis, hydrocele) and cutaneous findings (hemangioma)) has no precedent in *KDM5B* or *KDM5C* LoF literature and collectively suggests that homozygous loss of *KDM5A* may disrupt a broader set of developmental programs during embryogenesis than loss of its paralogs. These observations are consistent with the distinct spatiotemporal expression patterns and non-redundant epigenomic targets of *KDM5A* relative to *KDM5B* and *KDM5C*, as previously documented [13, 15] and underscore the importance of expanding the phenotypic framework of NEDEHC to accommodate multisystem involvement beyond the central nervous system.

The constellation of additional anomalies observed in our patient (hypothyroidism, pectus excavatum, talipes equinovarus, rocker-bottom feet, overriding second toe, developmental dysplasia of the hip, micropenis, hydrocele and hemangioma) extends the known phenotypic boundaries of NEDEHC. It suggests that biallelic *KDM5A* loss-of-function may give rise to a broader multisystem developmental syndrome than currently recognised. These findings enrich the variant and phenotypic landscape of *KDM5A*-related neurodevelopmental disorders and highlight the need for larger prospective cohorts to establish robust genotype-phenotype correlations and to clarify the basis of the phenotypic variability observed across reported cases.

## Supplementary information

Supplementary figures

## Data Availability

The data that support the findings of this study are available on request from the senior author, upon reasonable request. The data are not publicly available due to privacy or ethical restrictions.

## References

[1] KDM5A lysine demethylase 5A [Homo sapiens (human)], https://www.ncbi.nlm.nih.gov/gene/5927 2016.

[2] Kataria A, Tyagi S. Domain architecture and protein–protein interactions regulate KDM5A recruitment to the chromatin. Epigenetics. 2023;18:2268813. 10.1080/15592294.2023.2268813. PMC10578193 37838974

[3] Sheikhi M, et al. Prognostic importance of NUP98-rearrangements in acute myeloid leukemia: a systematic review and meta-analysis. Casp J Intern Med. 2024;15:579–88. 10.22088/cjim.15.4.579. PMC11444110 39359452

[4] Yang GJ, et al. Pharmacological inhibition of KDM5A for cancer treatment. Eur J Med Chem. 2021;226:113855. 10.1016/j.ejmech.2021.113855. 34555614

[5] Yang GJ, et al. The emerging role of KDM5A in human cancer. J Hematol Oncol. 2021;14:30. 10.1186/s13045-021-01041-1. PMC7888121 33596982

[6] El Hayek L, et al. KDM5A mutations identified in autism spectrum disorder using forward genetics. Elife. 2020;9:e56883. 10.7554/eLife.56883 PMC7755391 33350388

[7] Miller JN, Pearce DA. Nonsense-mediated decay in genetic disease: friend or foe? Mutat Res Rev Mutat Res. 2014;762:52–64. 10.1016/j.mrrev.2014.05.001. PMC4260155 25485595

[8] Campbell AE, et al. Compromised nonsense-mediated RNA decay results in truncated RNA-binding protein production upon DUX4 expression. Cell Rep. 2023;42:112642. 10.1016/j.celrep.2023.112642. PMC10592454 37314931

[9] Najmabadi H, et al. Deep sequencing reveals 50 novel genes for recessive cognitive disorders. Nature. 2011;478:57–63. 10.1038/nature10423. 21937992

[10] Silva IM, Rosenfeld J, Antoniuk SA, Raskin S, Sotomaior VS. A 1.5Mb terminal deletion of 12p associated with autism spectrum disorder. Gene. 2014;542:83–86. 10.1016/j.gene.2014.02.058. 24613754

[11] Han JY, Park J. Variable phenotypes of epilepsy, intellectual disability, and schizophrenia caused BY 12p13.33-p13.32 Terminal microdeletion in a Korean family: a case report and literature review. Genes. 2021;12. 10.3390/genes12071001 PMC8303811 34210021

[12] Szot JO, et al. A screening approach to identify clinically actionable variants causing congenital heart disease in exome data. Circ Genom Precis Med. 2018;11:e001978. 10.1161/CIRCGEN.117.001978. 29555671

[13] El Hayek, L et al. KDM5A mutations identified in autism spectrum disorder using forward genetics. Elife. 2020;9. 10.7554/eLife.56883 PMC7755391 33350388

[14] Kirtana R, Manna S, Patra SK. Molecular mechanisms of KDM5A in cellular functions: Facets during development and disease. Exp Cell Res. 2020;396:112314. 10.1016/j.yexcr.2020.112314. 33010254

[15] El Hayek L, et al. Disruption of the autism gene and chromatin regulator KDM5A alters hippocampal cell identity. Sci Adv. 2023;9:eadi0074. 10.1126/sciadv.adi0074. PMC10664992 37992166

[16] El Hayek, L et al. Monoallelic and biallelic KDM5A variants identified in patients with autism spectrum disorder. Human Genetics Genom Adv. 2026;7:100594. 10.1016/j.xhgg.2026.100594. PMC13090590 41889174

[17] Faundes V, et al. Histone lysine methylases and demethylases in the landscape of human developmental disorders. Am J Hum Genet. 2018;102:175–87. 10.1016/j.ajhg.2017.11.013. PMC5778085 29276005

[18] Lebon, S et al. Agenesis of the corpus callosum with facial dysmorphism and intellectual disability in sibs associated with compound heterozygous KDM5B variants. Genes. 2021;12. 10.3390/genes12091397 PMC8467522 34573379

[19] Borroto, MC et al. A genotype/phenotype study of KDM5B-associated disorders suggests a pathogenic effect of dominantly inherited missense variants. Genes. 2024;15. 10.3390/genes15081033 PMC11353349 39202393

[20] Wu PM, et al. Novel variations in the KDM5C gene causing X-linked intellectual disability. Neurol Genet. 2022;8:e646. 10.1212/NXG.0000000000000646. PMC8641966 34877407

[21] Lippa NC, Barua S, Aggarwal V, Pereira E, Bain JM. A novel de novo KDM5C variant in a female with global developmental delay and ataxia: a case report. BMC Neurol. 2021;21:358. 10.1186/s12883-021-02380-9. PMC8447699 34530748

[22] Terry, BK, Mahoney, A, Lee, BI, Secombe, J. Individuals with reported and novel KDM5C variants present with seizures, a feature recapitulated in a Drosophila model. Hum Mol Genet. 2026;15. 10.1093/hmg/ddag001 PMC12989173 41537560

