# Supplementary material for: Identification and characterisation of a novel homozygous KDM5A variant associated with severe axial hypotonia, seizures, and cardiac anomalies
Source: J Hum Genet. 2026 Aug 19;71(11):741–56. doi: 10.1038/s10038-026-01506-6 (PMC13695575; doi:10.1038/s10038-026-01506-6)
Supplement: Supplementary file 1 [file 10038_2026_1506_MOESM1_ESM.docx]

**Supp. Fig. 1.** Protein-protein interaction analysis of the KDM5A protein.

**Supp. Fig. 2.** Structural modelling of wild-type and mutant KDM5A proteins. (A) Predicted three-dimensional structure of the wild-type KDM5A protein. (B) Structural model of the truncated mutant KDM5A protein carrying the p.(Gln946Ter) variant. (C) Superimposition of the wild-type (blue) and mutant (yellow) KDM5A structures showing the structural differences caused by the truncation of the C-terminal region.

**Graphical abstract**
